# The Functions of the Board of Directors in Corporate Philanthropy: An Empirical Study From China

**DOI:** 10.3389/fpsyg.2022.850980

**Published:** 2022-05-24

**Authors:** Qi Pan, Zhangjie Huang

**Affiliations:** ^1^Institute of Service Engineering, Hangzhou Normal University, Hangzhou, China; ^2^School of Economics, Hangzhou Normal University, Hangzhou, China

**Keywords:** corporate philanthropy, board of directors, monitoring, consulting, environmental dynamism, board fault lines

## Abstract

As an important way for enterprises to fulfill social responsibility, corporate philanthropy (CP) has attracted much attention from the academic community. But there are still few well-targeted theoretical and empirical studies on what functions the board of directors (BOD) should perform to better fulfill philanthropic responsibilities. Taking this deficiency as a breakthrough, this study focuses on Chinese state-owned and private enterprises to analyze and test the functions performed by the BOD in CP. Based on the sample of Chinese A-share listed companies from 2008 to 2019, the empirical results show that the BOD of state-owned enterprises mainly performs a monitoring function in CP while that of private enterprises mainly performs a consulting function. The above findings remain valid when potential biases in the quantitative analysis are considered. Further research shows that environmental dynamism and board fault lines inhibit the performance of the above two functions. The contributions of the study include clarifying the functional characteristics of the BOD in CP and its influencing factors, revealing new theories to the formation mechanism of CP, which provide references for enterprises to optimize philanthropic decision-making. The limitation should also be emphasized that our findings are based only on Chinese contexts.

## Introduction

With the comprehensive advancement and rapid development of social welfare undertakings, corporate social responsibility (CSR) has become a significant issue of public concern. More and more enterprises are increasingly focused on practicing CSR. Many enterprises are diligently seeking ways to demonstrate social responsibility through corporate philanthropy (Erusalimsky et al., 2006; Luo et al., 2020; Jonawski, 2021). According to the 2019 Report on Chinese Charitable Donations, enterprises are the main source of charitable donations in China. More than 90% of enterprises have participated in charity work in different ways while enterprises’ donations accounted for more than 60% of the total.

Although CP has become an important means for such enterprises to practice CSR, the empirical studies needed to explore how to perform CP effectively are still lacking, especially regarding what functions the board of directors (BOD) should perform (Rao and Tilt, 2016; Boivie et al., 2021). In practice, due to the lack of reasonable philanthropic decision-making, enterprises have failed to establish a positive image in society through CP. Instead, unnecessary troubles can arise. In December 2020, minority shareholders filed a class action lawsuit against Kweichow Moutai over an external donation of 809 million RMB, and Kweichow Moutai was eventually forced to cancel the donation. Similarly, in April 2020, Vanke donated 200 million shares worth about 5.3 billion to the Tsinghua University Education Foundation. The decision was challenged by the employees of Vanke, who urged Tsinghua University to return the donation.

To avoid such embarrassment caused by donations, it is necessary to optimize the work of CP. This requires further exploration of the formation mechanism of CP, especially fully understanding the functions of the BOD (Krüger, 2009; Rao and Tilt, 2016; Zhuang et al., 2018), making full use of the BOD as the hub of enterprise’s management mechanism, formulating appropriate philanthropy plans to respond to the demands of stakeholders and ultimately improving CP satisfaction.

Although it is clear that the above-mentioned topics require further research, few studies have addressed them directly (Rao and Tilt, 2016). Some literature has examined the formation mechanism of CP from the perspective of the BOD, but most analyze the relationship between the characteristics of the BOD and CP. As a result, even if we find that a particular characteristic of the BOD affects CP, it is still not clear which function of the BOD works (Wang and Coffey, 1992; Williams, 2003; Gautier and Pache, 2015; Cha and Rew, 2018; Boivie et al., 2021; Endrikat et al., 2021). In fact, only by clearly identifying the functions of the BOD in CP can we better understand and improve the functions and eventually optimize and improve CP decision-making in a scientific way. Actually, this is also an important issue that has not been effectively explored in the current CSR research (Rao and Tilt, 2016; Bolourian et al., 2021).

In this study, we intend to improve these research deficiencies. Specifically, taking the monitoring and consulting function of the BOD as starting point, and considering the differences in the philanthropic motivations of enterprises with different property rights, this study develops a performance model of CP and uses it as a basis for inference to test the functions of the BOD of state-owned and private enterprises in CP. Based on the samples of Chinese A-share listed companies, the empirical results show that the BOD of state-owned enterprise (SOEs) mainly perform a monitoring function while that of private enterprises mainly perform a consulting function in CP. In addition, environmental dynamism and board fault lines inhibit the performance of these two functions.

The remainder of the study is structured as follows. In section “Literature Review and Hypothesis Development,” we review the prior research on the functions of the BOD and CP, propose the hypotheses. Section “Research Design” describes the data source, variables, and model. In section “Empirical Results and Analysis,” regression analysis is conducted to examine the hypotheses, followed by heterogeneity analysis and robustness checks. Section “Extension Analysis” tests the moderate effects of environmental dynamism and board fault lines. “Discussion” summarizes the contributions, proposes policy recommendations, future research, and conclusions.

## Literature Review and Hypotheses Development

### Literature Review

#### The Functions of the BOD

It refers to the roles, responsibilities, and tasks assumed by the BOD. As the core of a corporate governance and the principal entity of corporate decision-making, the BOD usually serves monitoring and consulting function, and their basic goal is to create sustainably growing value for shareholders (Schmidt and Brauer, 2006). As for the monitoring function, Fama and Jensen (1983) put forth monitor hypothesis based on agency theory, which holds that the main function of the BOD is to monitor, including hiring & dismissal of managers and determining managers’ compensation etc. Its purpose is to monitor the managers and protect the rights and interests of shareholders. As for the consulting function, the analyses based on resource dependence theory have revealed that the function of the BOD should be to provide advisory and suggestions to managers or to help establish connections with other organizations based on a comprehensive and in-depth understanding of the enterprise’s operating conditions and strategic positioning (Hillman and Dalziel, 2003), ultimately reducing the uncertainty of the external environment and improving the success rate of the company’s strategy implementation (Salancik and Pfeffer, 1978).

Since the functions of the BOD are largely invisible (Carter and Lorsch, 2003), most existing studies focus on the organization characteristics of the BOD and exploring whether certain characteristics effectively stimulates specific function. To reduce self-interested behavior of managers, the studies mainly examine the relationship between board structure, behavior requirements, incentive characteristics, and monitoring function. To improve consulting function of the BOD, the studies primarily focus on board members’ industry expertise, business background, and professional knowledge etc.

The above researches are beneficial for understanding the functions of the BOD. Different characteristics of the BOD are only external representations after all. Whether or how the functions of the BOD are effectively performed therefore remains unclear. As Golden and Zajac (2001) have pointed out, although people generally have high expectations to the BOD, it is still uncertain whether its functions are fulfilled. Taking this flaw as a breakthrough, relevant literature has further deepened and expanded mainly from two directions. First, some studies directly investigate the roles played by specific board members. For example, Liu et al. (2015) studied whether non-local independent directors play an advisory role in inter-province mergers and acquisitions. Zhu et al. (2021) examined the monitoring motives and its effects of non-controlling shareholder directors. Second, explore the functions of the BOD from the perspective of specific corporate practices. For example, Klarner et al. (2020) studied the functions of the BOD in corporate innovation. In more detail, there is also literature that specifically analyses the roles played by particular board members in a given corporate practice. For example, Lara et al. (2017) studied the monitoring function of female directors in the release of company accounting information.

#### Corporate Philanthropy

Many studies, whether considering CP as a dimension of CSR or focusing on the topic itself, have furnished fruitful and in-depth research on its antecedents (Gautier and Pache, 2015; Liket and Simaens, 2015; Cha and Rajadhyaksha, 2021).

Relevant research studied from four different perspectives. The first is analyzing the effect of CEO or other senior leaders on CP from the individual level. The main factors studied include gender, personality of the leader, professional background, social connections, discretion, and values (Campbell et al., 1999; Williams, 2003; Zhang et al., 2021). The second examines the factors that affect CP from the perspective of enterprise characteristics, which include corporate property rights, resources, size, debt ratio, advertising expenditure, geographic location, labor intensity, culture, R&D intensity and corporate history etc. (Zhang et al., 2010; Gao et al., 2011; Li et al., 2015a,b). The third explores from the perspective of corporate governance. The relevant literature mainly focuses on the composition of the BOD, remuneration, and incentives, the proportion of institutional investors and equity concentration etc. (Wang and Coffey, 1992; Su and Sauerwald, 2018). The fourth identifies the impact of macro environmental factors on CP, such as market system, taxation, and industry background (Brammer and Millington, 2004; Carroll and Joulfaian, 2005; Chih and Chen, 2009; Pan et al., 2017).

Summarizing the above two aspects of literature, we can find that fewer studies combine the functions of the BOD with CP, especially rarely examine the formation mechanism of CP from the functions of the BOD. Although some studies have tested the impact of the composition characteristics of the BOD on CP, there is still a long way to go before the functions of the BOD are clearly revealed. This is not only because the findings of many studies on the influence of board composition on CP remain inconsistent (Bolourian et al., 2021; Endrikat et al., 2021), but also because various structures and mechanisms of the BOD are only means serving the purpose of decision-making, which are not directly related to what function the BOD actually performs. Further, it provides an opportunity for this study to directly explore the functions of the BOD in CP.

### Research Hypothesis

#### The Functions of the BOD in CP

What functions does the BOD perform in CP? As far as Chinese enterprises are concerned, the coexistence of enterprises with different property rights during the transformation period has determined that state-owned and private enterprises would be in the spotlight (Wang and Qian, 2011). In view of the heterogeneity of the two types enterprises in CP orientation, and as the BOD should serve CP orientation, this study suggests that the functions of their BOD in CP are significantly different.

First, regarding SOEs, we infer that the BOD would position themselves in a monitoring role based on realistic considerations. Specifically, on one hand, SOEs should bear more social responsibilities including philanthropy (Zhang, 2013; Pan et al., 2015). The reason lies in the principle that social responsibilities shouldered by enterprises should match their rights. SOEs have more rights to development and resource use (Jin et al., 2014; Li and Cheong, 2019), which are manifested at least in the following facts. In terms of development, most of them are monopolies, through which they gain excessive profits (Huang, 2006; Wang and Yung, 2011). In terms of resource use, many SOEs expand their business by using more scarce economic resources, such as relying on bank credit rather than their own funds, and obtaining more quotas to become listed while enjoying more preferential policies including financial subsidies, land supply, and tax incentives etc. (Luo and Liu, 2009; Xu and Zhang, 2015). As SOEs have enjoyed the above privileges, they should accordingly shoulder more responsibilities including philanthropy.

On the other hand, due to owner absence and the lack of capital personalization, the excessive principal–agent chains of SOEs makes management team to weak supervision (Zheng et al., 2014; Hong et al., 2021). Coupled with the problem of information asymmetry, members of management team have the motivation to benefit themselves by using resources of SOEs to do more charity work. Existing studies have also revealed that the manager of SOEs have a strong desire to polish their personal images through charitable donations to pursue political promotion (Dai et al., 2014; Zhang et al., 2015).

In sum, SOEs must engage in CP for their privileges and inevitably encounter principal–agent problems at the same time. To avoid the loss of state-owned assets caused by charity works, the BOD is obliged to actively perform the monitoring function or take this as the function orientation.

While the BOD of SOEs positions itself in a monitoring role, given that competitiveness of monitoring and consulting functions (Armstrong et al., 2010; Faleye et al., 2011; Masulis et al., 2012; Boivie et al., 2021), it is not feasible to expect the BOD to do much work related to the consulting function. In fact, as stakeholders generally expect SOEs to be charitable, by nature, philanthropic responsibilities of SOEs is just a social response to stakeholder claims. In others words, when SOEs are subject to extensive pressure, stakeholders have actually set a framework for their philanthropic acts (Tan and Tang, 2016). At this point, there is insufficient demand for the consulting function of the BOD. And what is needed is immediate action rather than advisory. Thus we propose the following hypothesis:

***Hypothesis 1a**: The BOD of SOEs mainly performs monitoring functions in CP*.

Furthermore, for private enterprises to seek better development through donations, this study infers that the BOD will position themselves in a consulting role due to the practical needs of business operations. Specifically, although the private sector is a key component of Chinese economy, the implicit discrimination and the constraints placed on private enterprises have not been fully eliminated (Allen et al., 2005; Poncet et al., 2010; Lu et al., 2012). No form of private economy was allowed before 1987, and private enterprises were once regarded as enemies that exploited the people (Hong, 2004). Although private enterprises have been given new economic roles since reform and opening up, the public’s prejudices against private enterprises remains deep-seated. People believe that such enterprises are wealthy but uncharitable, think that all businessmen are dishonest, and believe that private enterprises have original sins. As a result, private enterprises have always come under harsh and suspicious scrutiny.

In order to enhance their legitimacy, it has become a natural and realistic choice for private enterprises to seek various kinds of social support. One option is to gain the favor of stakeholders by proactively fulfilling their social responsibilities. For example, they might establish political connections with the government through CP and then using the connections to reduce financing costs, obtain tax reliefs, obtain financial subsidies and other facilities (Zhong, 2007; Li et al., 2016). Li et al. (2015a,b) find that private enterprises may obtain debt financing by exchanging resources with the government through donations; the empirical study of Su and He (2010) also show that private enterprises obtain property rights protection through CP.

In summary, driven by interest, private enterprises in the transitional period will proactively allocate resources in donations. When private enterprises invest resources in charity, considering the complexity of the value effect of CP, they will inevitably enhance the effort of managing the philanthropic resources, such as brainstorming, weighing up the costs and benefits of charitable acts (Krulicky and Horak, 2021). To help CP achieve intended goals, as the core organization to facilitate enterprise development and operation management, the BOD has the responsibility to position itself in a consulting role.

Similarly, when the BOD of a private enterprise positions itself in the above-mentioned consulting role, given that the monitoring and consulting functions of the BOD are in competition (Armstrong et al., 2010; Faleye et al., 2011; Masulis et al., 2012; Boivie et al., 2021), it is less likely that the BOD will itself be in a monitoring role. In fact, as an important feature of the corporate organization, most private enterprises in China are family enterprises with blood relation (Gregory et al., 2000; Jiang et al., 2015). The interests of the owners and the manager are relatively consistent, and thus the principal–agent problem can be relieved to some extent. Moreover, different from owner absence of SOEs, the owner of a private enterprise can more easily exercise strict monitoring on external managers, which can restrain managers from seeking personal interests through philanthropy (Boateng et al., 2017). In short, the existence of the above conditions reduces the necessity of monitoring role played by the BOD in private enterprises. Hence, We formulate the following hypothesis.

***Hypothesis 1b**: The BOD of private enterprises mainly performs consulting functions in CP*.

#### Factors Affecting the Functions of the BOD in CP: Extensive Analysis

The functions of the BOD in CP is subject to certain conditions. The factors that affect the functions of the BOD are further identified as follows.

##### External Factors: Environmental Dynamism

As an important part of environmental uncertainty, environmental dynamism refers to the rate of environmental change and the degree of instability, which reflects the volatility and unpredictability of the external environment. It is reflected in an enterprise’s inaccurate perception of the changes in the behavior and needs of stakeholders. In a dynamic environment, the management work of an enterprise is more cumbersome, and there are high requirements for the processing of the decision-making information (Baum and Wally, 2003). This will potentially affect a series of corporate behaviors (Daft and Weick, 1984; Daft et al., 1988).

Specifically, the increase in environmental dynamism means that the BOD faces higher decision-making costs, should spend more energy on processing environmental information, and face greater difficulties in identifying the suitable programs, so the effect of the monitoring and consulting functions of the BOD will be greatly weakened. For SOEs, environmental dynamism facilitates managers to act for self-interest at the expense of enterprise resources. As an insider, managers have information advantages (Jensen and Meckling, 1976), and it is more difficult to monitor managers through the internal governance mechanism in this context. Accordingly, the consulting function of the BOD can also be constrained. For private enterprises, environmental dynamism weakens the capability of the BOD to make effective decisions based on adequate information. In the donation process, to achieve the goals of a private enterprise, careful arrangements must be made based on a large amount of environmental information. Such arrangements include consulting professional institutions, formulating donation plans, budgeting costs for donation before the implementation, managing the donation projects and controlling the costs during the process, and evaluating the donation projects after implementation (Zhong, 2007). Due to lack of or insufficient information, environmental dynamism makes it difficult for the BOD to make accurate judgments on the above issues, which will directly lead to difficulty in giving effective suggestions concerning donations. We thus propose the following hypotheses.

***Hypothesis 2a**: Environmental dynamism inhibits the monitoring function of the BOD of SOEs in CP*.

***Hypothesis 2b**: Environmental dynamism inhibits the consulting function of the BOD of private enterprises in CP*.

##### Internal Factors: Board Fault Lines

Although the previous sections have reasoned that the BOD plays a specific role in CP, in a strict sense, the BOD is not a solidified whole. When there are fault lines between the board members, the performance of its functions is bound to be affected. Fault lines refer to the invisible dividing lines between several homogeneous subgroups based on the single or multiple characteristic attributes of group members (Lau and Murnighan, 1998, 2005). Usually, people identify the people with similar characteristics and communicate with them, which leads to the existence of fault lines. As a result, team members will become more identified with their respective subgroups rather than the entire team, which intensifies conflicts within the team and reduces the trust and respect among team members. And team’s attention will ultimately turn from unified to splitting, weakening solidarity of the team (Li and Hambrick, 2005; Harrison and Klein, 2007; Bezrukova et al., 2009). Relevant evidences also show that, when fault lines exist in the BOD, the degree of engagement of the BOD will decrease (Zhou et al., 2015). The members will shorten the time of meeting and reduce the discussions on key issues of the company (Tuggle et al., 2010). All of these will result in weakening the willingness to monitor the company and provide resources (Xu et al., 2021), eventually lowering the performance of the directors in fulfilling the service functions of consultation and strategic decision-making (Crucke and Knockaert, 2016). In view of the above influence of the fault lines, we infer that the monitoring function of the BOD of SOEs and the consulting function of the BOD of private enterprises will be weakened in CP. Thus we propose the following hypotheses.

***Hypothesis 3a**: Board fault lines inhibit the monitoring function of the BOD of SOEs in CP*.

***Hypothesis3b**: Board fault lines inhibit the consulting function of the BOD of private enterprises in CP.*

## Research Design

### Data Sources and Research Samples

With reference to similar literature (Pan, 2018), the original samples are Chinese A-share listed firms on Shanghai and Shenzhen stock exchanges for the 2008–2019 period. The samples were screened according to the following rules: (1) excluding listed financial and insurance companies, (2) excluding ST and PT companies, (3) eliminating companies with severely incomplete data, and (4) excluding samples with obvious errors in donation data or with unidentifiable donation amount. The sample data come from the China Stock Market & Accounting Research Database (CSMAR). During the data collection process, if there were uncertain data need to be collected, professionals joined in the discussion to ensure reliability before finally determining the data value. After screening, our final sample of state-owned enterprises consists of 3,734 firm-years observations and that of private enterprises 3,535 firm-years observations. To reduce the influence of outliers on the research results, the continuous variables are winsorized at the 1 and 99% levels.

### Model Setting and Variable Definitions

Empirically, it is difficult to identify whether the BOD performs a monitoring or consulting function in CP as this is information or action that is hidden from researchers. Although it is difficult to find direct evidence of monitoring or consulting of the BOD, it is possible to infer from obvious external characteristics (Savova, 2021). By clarifying such external characteristics, the functions of the BOD can be inferred indirectly. Specifically, if the BOD does perform a certain function in CP, the economic value of donations should have been elevated as a result (Laing and Weir, 1999; Carter and Lorsch, 2003; Zhong, 2007). Based on the inference, this study constructs the following model to test Hypothesis 1:


(1) $$\begin{array}{c} {ROA}_{i,t}=\alpha_{0}+\alpha_{1}\times {CP}_{i,t-1}+\alpha_{2}\times {CP}_{i,t-1}\times \;\\ {\mathrm{Monitor}}_{i,t-1}+\alpha_{3}\times {CP}_{i,t-1}\times \\ {\mathrm{Consult}}_{i,t-1}+\alpha_{4}\times \mathrm{Control}+\varepsilon \end{array}$$

Consistent with Wang et al. (2008), return on total assets (ROA) is used as the proxy variable of enterprise performance. Corporate charitable donation is used as the proxy variable of CP, which is calculated by the ratio of enterprise’s total amount of donations to the main business revenue. With reference to the approach of Frye and Pham (2020), the discretionary accruals estimated by the modified Jones model are used as the indicator of the monitoring function (Monitor). The larger the value, the more obvious the monitoring function. Specifically, the calculation is done with the following model:


(2) $$\frac{TA_{i,t}}{\mathrm{Asse}t_{i,t-1}}=\alpha_{1}\frac{1}{\mathrm{Asse}t_{i,t-1}}+\alpha_{2}\frac{\Delta REV_{i,t}}{\mathrm{Asse}t_{i,t-1}}+\alpha_{2}\frac{PPE_{i,t}}{\mathrm{Asse}t_{i,t-1}}+\varepsilon_{i,t}$$

(3) $$\begin{array}{c} \;DA_{i,t}=\frac{TA_{i,t}}{\mathrm{Asse}t_{i,t-1}}-\\ \left(\overset{\wedge }{\;\alpha_{1}}\frac{1}{\mathrm{Asse}t_{i,t-1}}+\;\overset{\wedge }{\alpha_{2}}\frac{\Delta REV_{i,t}-\Delta REC_{i,t}}{\mathrm{Asse}t_{i,t-1}}+\;\overset{\wedge }{\alpha_{3}}\frac{PPE_{i,t}}{\mathrm{Asse}t_{i,t-1}}\right) \end{array}$$

Use Model (2) to run regressions by year and industry on the sample data, put the obtained regression coefficients into Formula (3), and then estimate discretionary accruals (*DA*). Among them, TA_*i*,*t*_ is the total accrual of sample *i* at the end of period *t*, Asset_i,*t*−1_ is the total assets of sample *i* at the end of period *t*−1, ΔREV_*i*,*t*_ is the change of the main business of sample *i* from period *t*−1 to period *t*. PPE_*i*,*t*_ is the original value of fixed assets of sample *i* at the end of period *t*, and ΔREC_*i*,*t*_ is the change of the receivable account of sample *i* from period *t*−1 to period *t*.

With reference to the approaches of Kim et al. (2014), Richardson (2006), and Zhu et al. (2015), corporate inefficient investment is obtained with the Richardson model and is used as the proxy variable for the consulting function of the BOD. The smaller the value, the more obvious the consulting function of the BOD. The model is as flows.


(4) $$\begin{array}{c} {\mathrm{Invest}}_{i,t}=\alpha_{0}+\alpha_{1}\times {\mathrm{Growth}}_{i,t-1}+\alpha_{2}\times \\ {\mathrm{Lev}}_{i,t-1}+\alpha_{3}\times {\mathrm{Cash}}_{i,t-1}+\alpha_{4}\times \\ {\mathrm{Age}}_{i,t-1}+\alpha_{5}\times {\mathrm{Asset}}_{i,t-1}+\alpha_{6}\times \\ {\mathrm{Return}}_{i,t-1}+\alpha_{7}\times {\mathrm{Invest}}_{i,t-1}+\varepsilon \end{array}$$

In model (4), “Invest” is the new investment of sample *i* in the current year, which is calculated by Invest = (capital expenditure+M&A costs−income from the sale of long-term assets−depreciation)/total assets. “Growth” is the growth rate of business revenue, “Cash” equals the enterprise’s cash and cash equivalents divided by total assets, “Age” is the enterprise’s age, “Asset” is the natural logarithm of the enterprise’s total assets at the end of the period, and “Return” is the annual stock return of the enterprise. Additionally, the year and industry effects are controlled in Model (4). The absolute residual obtained at the end is the indicator of the investment efficiency.

In addition, based on the existing literature (Wang et al., 2008; Choi and Wang, 2009; Lev et al., 2010; Wang and Qian, 2011; Brammer and Millington, 2015; Pan, 2018), control variables in the hypothesis testing model are employed as follows: (1) enterprise size ( Size ), defined as the natural logarithm of the company’s assets at the end of the period. (2) Enterprise debt ratio ( Lev ), defined as the ratio of the enterprise’s liabilities to assets. (3) Enterprise age ( Age ), defined as the natural logarithm of the enterprise’s years of age+1. (4) Enterprise growth opportunity ( Growth ), defined as the growth rate of the enterprise’s sales. (5) Enterprise risk ( Beta ), measured as the value of β . (6) Enterprise resources ( Res ), defined as the natural logarithm of the enterprise’s cash equivalents. (7) Advertisement ( AD ), defined as the ratio of sales expenses to operating income. (8) The industry (Industry), classified according to the latest classification of the China Securities Regulatory Commission in 2012. (9) The year (Year).

## Empirical Results and Analysis

### Descriptive Statistics

As shown in Tables 1, 2, the sample sizes of state-owned and private enterprises in this study are 3,743 and 3,535, respectively. The mean value of the returns on total assets (ROA) of the state-owned and private enterprises are 0.038 and 0.047, respectively. The performance of the private enterprises is slightly better than that of SOEs. In addition, CP of the private enterprises are greater than those of the SOEs with mean values of 0.277 and 0.190, respectively. Furthermore, the monitoring (Monitor) and consulting (Consult) function of the state-owned and private enterprises are relatively close. The monitoring function (Monitor) of the two are 0.008 and 0.010, respectively, and the consulting function of the two (Consult) are 0.033 and 0.038, respectively. For the control variables, our descriptive statistics are generally consistent with historical literature (Wang et al., 2008; Choi and Wang, 2009; Lev et al., 2010; Wang and Qian, 2011; Pan, 2018).

**Table 1:** Descriptive statistics of the main variables (SOEs).

Variable	Mean	SD	Min	P25	P50	P75	Max	Size
Roa	0.038	0.036	−0.026	0.014	0.031	0.055	0.129	3,743
CP × Monitor	0.001	0.020	−0.111	−0.002	0.000	0.004	0.140	3,743
CP × Consult	0.007	0.014	0.000	0.001	0.002	0.007	0.131	3,743
CP	0.190	0.241	0.011	0.036	0.091	0.234	1.027	3,743
Monitor	0.008	0.060	−0.108	−0.031	0.007	0.042	0.136	3,743
Consult	0.033	0.032	0.002	0.011	0.023	0.043	0.127	3,743
Beta	1.156	0.232	0.713	1.004	1.153	1.302	1.630	3,743
Growth	0.130	0.226	−0.224	−0.015	0.101	0.237	0.724	3,743
Size	22.896	1.150	20.911	21.941	22.858	23.822	24.764	3,743
Lev	0.530	0.180	0.156	0.396	0.551	0.676	0.790	3,743
Ad	0.055	0.062	0.003	0.015	0.034	0.070	0.279	3,743
Res	20.716	1.207	18.451	19.785	20.712	21.623	22.703	3,743
Age	2.663	0.388	1.609	2.398	2.773	2.944	3.178	3,743

**Table 2:** Descriptive statistics of the main variables (private enterprises).

Variable	Mean	SD	Min	P25	P50	P75	Max	Size
Roa	0.047	0.040	−0.026	0.018	0.042	0.072	0.129	3,535
CP × Monitor	0.003	0.026	−0.011	−0.004	0.001	0.007	0.140	3,535
CP × Consult	0.022	0.039	0.000	0.002	0.007	0.023	0.281	3,535
CP	0.277	0.293	0.011	0.068	0.160	0.371	1.027	3,535
Monitor	0.010	0.062	−0.108	−0.031	0.007	0.047	0.136	3,535
Consult	0.038	0.034	0.002	0.012	0.027	0.051	0.127	3,535
Beta	1.179	0.250	0.713	1.006	1.181	1.354	1.630	3,535
Growth	0.163	0.242	−0.224	0.004	0.128	0.280	0.724	3,535
Size	22.353	0.901	20.911	21.688	22.297	22.900	24.764	3,535
Lev	0.432	0.177	0.156	0.292	0.428	0.561	0.790	3,535
Ad	0.083	0.080	0.003	0.027	0.051	0.109	0.279	3,535
Res	20.124	1.018	18.451	19.385	20.088	20.810	22.703	3,535
Age	2.286	0.457	1.609	1.946	2.197	2.639	3.178	3,535

### Regression Results

To ensure the reliability of the research results, after F, LM, and Hausman tests, the results indicate that a fixed effect model should be used. In the meantime, to reduce the deviation of the regression results, the standard errors are corrected by the Driscoll–Kraay method while cluster-robust standard error correction is carried out at the company level. During the test, the control variables are added first followed by the core variables. The regression results are shown in Table 3.

**Table 3:** Regression results of the functions of the BOD in CP.

	State-owned enterprises				Private enterprises			
	1	2	3	4	5	6	7	8
Constant	−0.057^***^ (−2.941)	−0.059^***^ (−3.011)	−0.040^**^ (−2.159)	−0.042^**^ (−2.225)	−0.098^***^ (−3.698)	−0.097^***^ (−3.779)	−0.058^**^ (−2.290)	−0.057^**^ (−2.339)
Beta	−0.000 (−0.173)	−0.001 (−0.196)	−0.002 (−0.616)	−0.002 (−0.634)	−0.013^***^ (−4.925)	−0.013^***^ (−4.991)	−0.012^***^ (−4.698)	−0.012^***^ (−4.752)
Growth	0.031^***^ (14.940)	0.030^***^ (14.743)	0.033^***^ (16.079)	0.032^***^ (15.933)	0.037^***^ (14.814)	0.036^***^ (14.279)	0.037^***^ (15.507)	0.037^***^ (15.004)
Size	0.001 (0.598)	0.001 (0.590)	−0.001 (−0.810)	−0.001 (−0.811)	0.002 (1.461)	0.002 (1.402)	−0.001 (−0.599)	−0.001 (−0.707)
Lev	−0.110^***^ (−19.662)	−0.110^***^ (−19.653)	−0.100^***^ (−18.226)	−0.100^***^ (−18.189)	−0.102^***^ (−17.564)	−0.102^***^ (−17.822)	−0.088^***^ (−15.742)	−0.089^***^ (−16.020)
Ad	−0.033^*^ (−1.806)	−0.035^*^ (−1.877)	−0.025 (−1.387)	−0.026 (−1.443)	−0.011 (−0.659)	−0.008 (−0.475)	0.001 (0.071)	0.005 (0.296)
Res	0.006^***^ (5.145)	0.006^***^ (5.126)	0.007^***^ (6.090)	0.007^***^ (6.073)	0.007^***^ (6.757)	0.007^***^ (6.879)	0.008^***^ (7.758)	0.008^***^ (7.919)
Age	−0.002 (−1.057)	−0.002 (−0.964)	−0.001 (−0.653)	−0.001 (−0.570)	−0.002 (−0.907)	−0.002 (−0.794)	−0.000 (−0.185)	−0.000 (−0.065)
CP	0.014^***^ (4.650)	0.013^***^ (3.531)	0.012^***^ (4.381)	0.011^***^ (3.173)	0.008^***^ (3.736)	0.015^***^ (5.665)	0.007^***^ (3.208)	0.015^***^ (5.659)
Consult		0.042^**^ (1.976)		0.032 (1.577)		0.053^**^ (2.568)		0.056^***^ (2.860)
Monitor			0.082^***^ (7.738)	0.081^***^ (7.670)			0.082 (0.797)	0.067 (0.646)
CP × Consult		0.002 (0.032)		0.015 (0.250)		−0.101^***^ (−4.402)		−0.115^***^ (−5.364)
CP × Monitor			0.089^***^ (2.682)	0.090^***^ (2.703)			0.005 (0.618)	0.006 |(0.786)
Industry	Yes	Yes	Yes	Yes	Yes	Yes	Yes	Yes
Year	Yes	Yes	Yes	Yes	Yes	Yes	Yes	Yes
*N*	3,743	3,743	3,743	3,743	3,535	3,535	3,535	3,535
*R* ^2^	0.418	0.418	0.470	0.470	0.333	0.352	0.398	0.420

^*^, ^**^, ^***^ represent significant at the 10, 5, and 1% significance level, respectively. *t*-values are provided in parentheses.

In the eight sets of regression results shown in Table 3, the regression coefficients of CP are all significantly positive, which indicates that CP can improve the enterprises’ performance. For SOEs, when the interaction terms of CP × Consult and CP × Monitor are added, the regression coefficient of CP × Monitor is 0.089, which is significant at the 1% level, while the regression coefficient of CP × Consult is not. When the two are put into the regression model at the same time, the regression coefficient of CP × Monitor is 0.090, which is still significant at the 1% level. Similarly, the regression coefficient of CP × Consult is still not significant. This shows that the performance of CP of SOEs has been improved under the influence of the monitoring function of the BOD. In other words, Hypothesis 1-a is empirically supported.

For private enterprises, when the interaction terms of CP × Consult and CP × Monitor are added, the regression coefficient of CP × Consult is −0.101, which is significant at the 1% level, while the regression coefficient of CP × Monitor is not. When the two are put into the regression model at the same time, the regression coefficient of CP × Consult is −0.115, which is still significant at the 1% level. Similarly, the regression coefficient of CP × Monitor is still not significant. This shows that the performance of CP of private enterprises has been improved under the influence of the monitoring function of the BOD. That is, Hypothesis 1-b is empirically supported.

### Robustness Test

#### Sample Selection Bias Test

In the empirical analysis of this study, there may be sample selectivity bias. Specifically, (1) the research objects of this study are the enterprises that have participated in donations, however, not all enterprises have donated. It is also possible that some enterprises have donated but have not disclosed. The fact that only the companies participating in donations are selected for research could lead to sample selection bias. (2) Even for the enterprises that have participated in donations, some samples have been deleted due to missing data, which also causes sample selection bias. For the second case, we compared the actual research sample with the initial sample and found that the kernel density distribution curves of corporate donations in the two samples basically overlap, which shows that the actual research sample can replace the initial sample.

For the first case, with reference to the approach of Pan et al. (2015), Heckman two-step estimation is adopted. In the first stage, run a probit regression, and it’s dependent variable is whether the enterprise has participated in donations in the previous year. After obtaining the inverse Mills ratio (denoted by *λ*), put it into Model (1) to alleviate the influence of sample selection bias. Table 4 presents the regression results. As shown in Table 4, after adding the *λ* variable, the signs and significance of the regression coefficients of CP × Consult and CP × Monitor, for both state-owned and private enterprises, are the same as in Table 3, and the research conclusions remain unchanged. In addition, the regression coefficients of *λ* itself are not significant.

**Table 4:** Regression results of the functions of the BOD in CP. (Heckman Second-stage).

	State-owned enterprises				Private enterprises			
	1	2	3	4	5	6	7	8
Constant	−0.088^***^ (−4.635)	−0.088^***^ (−4.650)	−0.062^***^ (−3.355)	−0.062^***^ (−3.368)	−0.127^***^ (−5.150)	−0.124^***^ (−5.096)	−0.103^***^ (−4.308)	−0.099^***^ (−4.199)
Beta	−0.010^***^ (−2.997)	−0.010^***^ (−2.974)	−0.012^***^ (−3.637)	−0.012^***^ (−3.609)	−0.017^***^ (−4.872)	−0.017^***^ (−4.812)	−0.016^***^ (−4.597)	−0.015^***^ (−4.528)
Growth	0.032^***^ (8.921)	0.032^***^ (8.683)	0.034^***^ (9.671)	0.033^***^ (9.389)	0.039^***^ (8.800)	0.038^***^ (8.476)	0.039^***^ (9.087)	0.037^***^ (8.735)
Size	0.003^**^ (2.504)	0.003^**^ (2.495)	0.001 (1.003)	0.001 (0.994)	−0.001 (−0.556)	−0.001 (−0.633)	−0.003^*^ (−1.926)	−0.003^**^ (−2.085)
Lev	−0.108^***^ (−21.916)	−0.109^***^ (−21.885)	−0.099^***^ (−20.252)	−0.099^***^ (−20.227)	−0.099^***^ (−16.797)	−0.100^***^ (−17.187)	−0.087^***^ (−15.167)	−0.089^***^ (−15.622)
Ad	−0.017 (−1.171)	−0.017 (−1.146)	−0.010 (−0.693)	−0.009 (−0.654)	0.011 (0.822)	0.011 (0.868)	0.019 (1.511)	0.020 (1.591)
Res	0.005^***^ (4.478)	0.005^***^ (4.469)	0.006^***^ (5.454)	0.006^***^ (5.440)	0.012^***^ (9.201)	0.012^***^ (9.203)	0.012^***^ (9.908)	0.012^***^ (9.959)
Age	−0.002 (−0.796)	−0.002 (−0.747)	−0.001 (−0.596)	−0.001 (−0.535)	−0.000 (−0.183)	−0.000 (−0.038)	0.001 (0.392)	0.001 (0.590)
CP	0.010^***^ (2.949)	0.008^*^ (1.799)	0.008^**^ (2.397)	0.005 (1.198)	0.010^***^ (3.535)	0.021^***^ (6.109)	0.011^***^ (3.681)	0.023^***^ (6.718)
*λ*		−0.008 (−0.947)	−0.009 (−0.975)	−0.010 (−1.168)	–	−0.000 (−0.030)	−0.002 (−0.188)	0.005 (0.548)
Consult		0.011 (0.406)		0.012 (0.433)		0.114^***^ (3.688)		0.130^***^ (4.393)
Monitor			0.095^***^ (6.787)	0.094^***^ (6.756)			0.129 (0.906)	0.085 (0.604)
CP × Consult		0.045 (0.631)		0.062 (0.894)		−0.165^***^ (−5.703)		−0.184^***^ (−6.627)
CP × Monitor			0.113^***^ (3.157)	0.116^***^ (3.225)			0.002 (0.153)	0.005 (0.495)
Industry	Yes	Yes	Yes	Yes	Yes	Yes	Yes	Yes
Year	Yes	Yes	Yes	Yes	Yes	Yes	Yes	Yes
*N*	3,179	3,179	3,179	3,179	2,977	2,977	2,977	2,977
*R* ^2^	0.418	0.418	0.470	0.470	0.333	0.352	0.398	0.420

#### Other Robustness Tests

To ensure the reliability of the aforementioned research conclusions, other robustness tests have also been conducted.

First, the explanatory variables and the explained variable are measured by alternative method. It mainly includes: (1) The ratio of an enterprise’s total donations to the total assets is used to measure the intensity of the enterprise’s donations. (2) Adjust the focus variables of concern with the industry average on a yearly basis. (3) The three-year moving average of ROA is used as the proxy variable for corporate performance.

Second, re-test through changing the samples. It includes: (1) Considering that the levels of enterprise donations during major disasters are significantly higher than in other ordinary years, with reference to Du et al. and Pan et al., the observations in 2010 (Qinghai earthquake) and 2013 (Yushu earthquake) are eliminated from the sample (Du et al., 2014; Pan et al., 2017). (2) Only when corporate donations exceed a certain amount do they need the approval of the BOD or should they be submitted to the authority for approval. As requirements of listed companies and regulations of various government set the threshold of donations at 100,000 RMB, to highlight the enterprises’ true willingness of donations, observations that donated less than 100,000 RMB are excluded. (3) The values of the continuous variables are winsorized at the 2–5% quantiles.

Third, consider the influence of more control variables. To further eliminate the interference of missing variable bias and overcome the endogenous problems caused by the effects of the individuals and the industries, high-dimensional fixed effect estimation is adopted. Specifically, the interaction terms of the enterprise’s province and industry with the year are added in the regression model.

In the results of the above regression test, the signs of the core variables remain unchanged, and the significance level is at least 95%, which indicate that the aforementioned conclusions are robust.

## Extension Analysis

To further identify the dependent conditions of the functions of the BOD in CP, further empirical test is conducted on the moderating effect of environmental dynamism and board fault lines.

### Environmental Dynamism

To test hypotheses 2, the following regression model is established:


(5) $$\begin{array}{c} {ROA}_{i,t}=\alpha_{0}+\alpha_{1}\times {CP}_{i,t-1}+\alpha_{2}\times {CP}_{i,t-1}\times \\ {\mathrm{Monitor}}_{i,t-1}\times ED+\alpha_{3}\times {CP}_{i,t-1}\times \\ {\mathrm{Consult}}_{i,t-1}\times ED+\alpha_{4}\times \mathrm{Control}+\varepsilon \end{array}$$

In Model (5), environmental dynamism (ED) is the moderator variable. With reference to the approach of Ghosh and Olsen (2009), ED is calculated by taking the company’s sales revenue in years *t*, *t*−1, *t*−2, *t*−3, and *t*−4 as the dependent variable and running autoregressions with 5, 4, 3, 2, and 1 as independent variables. The standard errors of the regression coefficients of the model are divided by the mean value of the company’s 5-year sales, and the obtained value is taken as the proxy variable. The larger the value, the stronger the environmental dynamism. The regression results are shown in Table 5.

**Table 5:** Regression results of the moderating impact of environmental dynamism.

	State-owned enterprises			Private enterprises		
	1	2	3	4	5	6
Constant	−0.037^*^ (−1.863)	−0.019 (−1.007)	−0.021 (−1.125)	−0.053^**^ (−2.116)	−0.020 (−0.813)	−0.025 (−1.023)
Beta	−0.001 (−0.199)	−0.002 (−0.611)	−0.002 (−0.625)	−0.013^***^ (−5.089)	−0.012^***^ (−4.830)	−0.012^***^ (−4.847)
Growth	0.022^***^ (11.310)	0.025^***^ (12.842)	0.024^***^ (12.707)	0.024^***^ (9.304)	0.027^***^ (11.055)	0.027^***^ (10.646)
Size	−0.000 (−0.237)	−0.002 (−1.574)	−0.002 (−1.499)	−0.001 (−0.462)	−0.003^**^ (−2.181)	−0.003^**^ (−2.197)
Lev	−0.110^***^ (−20.224)	−0.101^***^ (−18.760)	−0.101^***^ (−18.803)	−0.105^***^ (−18.797)	−0.092^***^ (−16.623)	−0.092^***^ (−16.860)
Ad	−0.026 (−1.418)	−0.018 (−0.982)	−0.018 (−1.011)	−0.001 (−0.055)	0.005 (0.325)	0.010 (0.629)
Res	0.005^***^ (4.753)	0.006^***^ (5.733)	0.006^***^ (5.663)	0.007^***^ (7.173)	0.008^***^ (7.811)	0.008^***^ (8.164)
Age	0.000 (0.007)	0.001 (0.270)	0.001 (0.322)	0.002 (1.070)	0.003 (1.523)	0.003 (1.480)
CP	0.012^***^ (3.182)	0.011^***^ (4.012)	0.010^***^ (2.930)	0.011^***^ (4.319)	0.006^***^ (2.866)	0.011^***^ (4.318)
ED	0.042^***^ (7.240)	0.042^***^ (8.167)	0.039^***^ (7.169)	0.056^***^ (10.061)	0.054^***^ (10.347)	0.045^***^ (8.266)
Consult	0.036^*^ (1.730)		0.028 (1.380)	0.019 (0.943)		0.025 (1.276)
Monitor		0.076^***^ (7.295)	0.075^***^ (7.233)		0.059 (0.510)	0.029 (0.249)
CP × Consult	−0.073 (−0.916)		−0.066 (−0.884)	−0.115^***^ (−4.422)		−0.137^***^ (−5.508)
ED × CP × Consult	0.367 (1.390)		0.381 (1.546)	0.493^***^ (2.913)		0.559^***^ (3.368)
CP × Monitor		0.159^***^ (3.227)	0.160^***^ (3.248)		0.005 (0.595)	0.008 (0.892)
ED × CP × Monitor		−0.350^**^ (−2.057)	−0.344^**^ (−2.028)		0.032 (0.315)	−0.001 (−0.007)
Industry	Yes	Yes	Yes	Yes	Yes	Yes
Year	Yes	Yes	Yes	Yes	Yes	Yes
*N*	3,743	3,743	3,743	3,535	3,535	3,535
*R* ^2^	0.427	0.472	0.475	0.361	0.395	0.426

For SOE, environmental dynamism is taken as the moderator variable, and the test is conducted by using it to form interaction terms with CP × Monitor and CP × Consult. In the results, the regression coefficients of ED × CP × Monitor are −0.350 and −0.344, respectively, and both are significant at the 5% level. Given that the monitoring function of the BOD of SOEs promotes the enterprise’s performance, the results show that, when the effect of environmental dynamism is considered, its monitoring function is weakened, which validates Hypothesis 2-a. For private enterprises, when putting the interaction term of ED × CP × Consult into the model, its regression coefficient is 0.493 and significant at the 1% level. After adding it together with ED × CP × Monitor, the regression coefficient of DE × CP × Consult is 0.559, which is still significant at the 1% level. Given that the consulting function of the BOD of private enterprises promotes the enterprise’s performance, the results show that the effect of environmental dynamism inhibits its consulting function, which is consistent with the proposition of Hypothesis 2-b.

Based on the above results, we can conclude that environmental dynamism is a constraint condition for performing the functions of the BOD in CP.

### Board Fault Lines

To test hypotheses 3, the following regression model is established:


(6) $$\begin{array}{c} {ROA}_{i,t}=\alpha_{0}+\alpha_{1}\times {CP}_{i,t-1}+\alpha_{2}\times {CP}_{i,t-1}\times \\ {\mathrm{Monitor}}_{i,t-1}\times FL+\alpha_{3}\times {CP}_{i,t-1}\times \\ {\mathrm{Consult}}_{i,t-1}\times FL+\alpha_{4}\times \mathrm{Control}+\varepsilon \end{array}$$

In model (6), FL is board fault lines. With reference to the approaches of Tuggle and other studies, board fault lines are calculated with five indicators as the basis: gender, age, education background, independent director or not, and length of tenure (Tuggle et al., 2010). The method is as follows. According to the formula proposed by Thatcher et al., the ratio of the sums of squares of the subgroups’ characteristics to that of the overall characteristics of the BOD is taken as the intensities of the fault lines (Thatcher et al., 2003). The calculation is done as below:


(7) $$FL_{g}=\frac{\sum_{j=1}^{q}\sum_{k=1}^{2}n_{k}^{g}{\left(\bar{x_{j,k}}-\bar{x_{j}}\right)}^{2}}{\sum_{j=1}^{q}\sum_{k=1}^{2}\sum_{i=1}^{n_{k}^{g}}{\left(x_{i,j,k}-\bar{x_{j}}\right)}^{2}}$$

$$FL_{i,t}=\max\left(FL_{g}\right)$$

First, the sum of squares of a particular characteristic of a subgroup is calculated with Formula (7). Next, the sum of squares of the same characteristics of the BOD is calculated. Calculate the intensity of fault line for all characteristics of the subgroup in turn, and the maximum value among all subgroups is taken as the measured value of the board fault line intensity. Among them, FL*_g_* denotes the fault line intensity under the g-th division method where *g* denotes the classification method, *n* denotes the number of board members, *j* denotes a certain characteristic of the board members, *q* denotes the total number of characteristics investigated, $x_{i,j,k}$ denotes the value of characteristic *j* of director *i* in subgroup *k*, $\bar{x_{j,k}}$ denotes the mean value of characteristic *j* of subgroup k’s board, $\bar{x_{j}}$ is the mean value of characteristic *j* of all members of the board, and $n_{k}^{g}$ denotes the number of members in subgroup *k* of the BOD under classification method *g*. The value range of board fault line ( FL ) is [0, 1]. The closer the value is to 1, the higher the intensity of the fault line. The regression results are shown in Table 6.

**Table 6:** Regression results of the moderating impact of board fault lines.

	State-owned enterprises			Private enterprises		
	1	2	3	4	5	6
Constant	−0.047^**^ (−2.234)	−0.028 (−1.411)	−0.031 (−1.543)	−0.082^***^ (−3.072)	−0.044^*^ (−1.659)	−0.043^*^ (−1.703)
Beta	−0.002 (−0.548)	−0.003 (−1.097)	−0.003 (−1.131)	−0.013^***^ (−4.791)	−0.012^***^ (−4.459)	−0.011^***^ (−4.432)
Growth	0.030^***^ (13.380)	0.034^***^ (15.038)	0.032^***^ (14.632)	0.035^***^ (13.457)	0.037^***^ (14.800)	0.035^***^ (13.958)
Size	−0.000 (−0.024)	−0.002 (−1.421)	−0.002 (−1.318)	0.003 (1.562)	−0.001 (−0.489)	−0.001 (−0.502)
Lev	−0.109^***^ (−18.545)	−0.099^***^ (−16.973)	−0.099^***^ (−17.205)	−0.102^***^ (−17.588)	−0.087^***^ (−15.015)	−0.089^***^ (−15.820)
Ad	−0.026 (−1.287)	−0.019 (−0.939)	−0.017 (−0.870)	−0.005 (−0.285)	0.001 (0.074)	0.008 (0.491)
Res	0.007^***^ (5.458)	0.008^***^ (6.376)	0.008^***^ (6.305)	0.007^***^ (6.346)	0.007^***^ (7.075)	0.008^***^ (7.270)
Age	−0.003 (−1.314)	−0.002 (−1.027)	−0.002 (−1.017)	−0.002 (−0.988)	−0.001 (−0.285)	−0.000 (−0.157)
CP	0.021^***^ (5.720)	0.012^***^ (3.903)	0.020^***^ (5.576)	0.009^***^ (2.858)	0.006^***^ (2.588)	0.008^***^ (2.781)
FL	−0.008^**^ (−2.377)	−0.007^**^ (−2.104)	−0.007^**^ (−2.023)	−0.011^***^ (−2.613)	−0.007 (−1.634)	−0.011^***^ (−2.751)
Consult	0.087^***^ (3.891)		0.083^***^ (3.715)	−0.001 (−0.056)		0.000 (0.008)
Monitor		0.102^***^ (10.585)	0.102^***^ (10.720)		0.129^***^ (9.953)	0.131^***^ (10.343)
CP × Consult	−0.004^*^ (−1.814)		−0.005^**^ (−2.316)	−0.009^***^ (−6.875)		−0.010^***^ (−8.089)
FL × CP × Consult	−0.011 (−0.220)		−0.001 (−0.025)	0.454^***^ (5.593)		0.487^***^ (6.243)
CP × Monitor		1.072^**^ (2.237)	1.079^**^ (2.200)		0.038 (0.563)	−0.007 (−0.123)
FL × CP × Monitor		−1.133^**^ (−2.285)	−1.133^**^ (−2.236)		0.010 (0.308)	0.038 (1.292)
Industry	Yes	Yes	Yes	Yes	Yes	Yes
Year	Yes	Yes	Yes	Yes	Yes	Yes
*N*	3,060	3,060	3,060	3,326	3,326	3,326
*R* ^2^	0.434	0.489	0.497	0.372	0.393	0.439

For SOEs, board fault line (FL) is taken as the moderator variable, and the test is conducted by using it to form interaction terms with CP × Consult and CP × Monitor. In the regression results, the regression coefficients of FL × CP × Monitor are all valued −1.133 and are all significant at the 5% level. The results show that, when the effect of board fault line is considered, the monitoring function of the BOD of SOEs is weakened. For state-owned enterprises, using board fault line (FL) as the moderator variable again, the regression coefficients of FL × CP × Consult are 0.454 and 0.487, and both are significant at the 1% level. It can be judged that board fault line weakens the consulting function of private enterprises of the BOD in CP. The above empirical findings are all consistent with the previous inferences and thus the hypotheses 3-a and 3-b were all tested. And it is reasonable to believe that board fault lines hinders the function of the BOD in CP.

## Discussion

As CP becomes an important means for companies to fulfil their social responsibility in China, this paper argues that in order to better respond to stakeholders, the focus needs to be on the functions of the BOD.

### Theoretical Contributions

While well-targeted discussions and tests of the function of the BOD are still lacking in CP research, this study has made up for such deficiencies.

The main contributions of this study are as follows. First, based on the empirical evidence from Chinese listed companies, this study clarifies the function orientation of the BOD in CP. This not only expands the understanding of the formation mechanism of corporate philanthropy, but also provides a theoretical reference for guiding enterprises to formulate appropriate philanthropy programs. Second, based on environmental dynamism and board fault lines, this study has identified the boundary conditions. This provides a clearer foundation for the actions to optimize or improve the function of the BOD in a more explicit approach and, in turn, to enhance the effectiveness of corporate philanthropic programs. Third, the study examines the heterogeneity of the functions of the BOD in CP between state-owned and private enterprises. It enriches the research on heterogeneity of CP and provides new insight for accurately grasping the qualitative characteristics of CP.

### Managerial Contributions

Our findings have important implications for companies and policymakers. First, to improve the financial effect of CP, the BOD should include charitable donations in the key decision-making agenda, especially focusing on and consolidating the functions of the BOD. Second, to avoid the misalignment of the function of the BOD in CP, SOEs can focus on the monitoring function when improving the governance mechanism while private enterprises can focus on the consulting function. Third, special attention should be paid to the impact of environmental dynamics and board fault lines on the functions the BOD in CP. In order to reduce the impact of environmental dynamics, enterprises can make their donation work more detailed through refined management so as to provide information to support the accurate decision-making of the BOD. To reduce the impact of board fault lines, the diversity of members in terms of their functional backgrounds, educational levels, and other cognitive characteristics should be fully considered when appointing board members, so as to reduce differences and enhance communication between board members, and ultimately enhance the synergy of decision-making.

### Limitation and Future Research

As with any other research, several limitations of this study could be improved. First, although the results from the sample provide meaningful insights into the functions of the BOD in CP, this study includes only Chinese A-share listed companies, which have a unique institutional and cultural background. Future studies need to applicate the model in other contexts in order to enhance the efficacy of the model. Second, the study focuses only on corporate charitable donation. However, there are other forms of fulfilling CSR. Future studies could consider the functions of the board of directors in other forms of CSR.

## Conclusion

To infer the functions of the BOD in CP, we started from the impact of the BOD on donation performance. Based on Chinese A-share listed companies that have participated in donations from 2008 to 2019 as the samples, the empirical findings show that the BOD of SOEs mainly perform monitoring function while that of private enterprises mainly perform consulting function. Furthermore, the extension analysis shows that the functions of the BOD is restricted by environmental dynamism and its own fault lines.

## Data Availability Statement

The data sets presented in this study can be found in online repositories. The names of the repository/repositories and accession number(s) can be found in the article/Supplementary Material.

## Author Contributions

QP: conceptualization, methodology, validation, writing-original draft preparation, and writing-review and editing. ZH: software, validation, and writing-review and editing. All authors contributed to the article and approved the submitted version.

## Funding

This research was funded by “Zhejiang Province Philosophy and Social Science Program, grant number 20NDJC168YB”; “Zhejiang Province University Major Humanities and Social Sciences Research Projects, grant number 2021GH006.”

## Conflict of Interest

The authors declare that the research was conducted in the absence of any commercial or financial relationships that could be construed as a potential conflict of interest.

## Publisher’s Note

All claims expressed in this article are solely those of the authors and do not necessarily represent those of their affiliated organizations, or those of the publisher, the editors and the reviewers. Any product that may be evaluated in this article, or claim that may be made by its manufacturer, is not guaranteed or endorsed by the publisher.

## Supplementary Material

The Supplementary Material for this article can be found online at: https://www.frontiersin.org/articles/10.3389/fpsyg.2022.850980/full#supplementary-material

## Abbreviations

CP: Corporate philanthropy
BOD: Board of directors
SOEs: State-owned enterprises
